# Involving patients in HTA activities at local level: a study protocol based on the collaboration between researchers and knowledge users

**DOI:** 10.1186/1472-6963-12-14

**Published:** 2012-01-16

**Authors:** Marie-Pierre Gagnon, Johanne Gagnon, Michèle St-Pierre, François-Pierre Gauvin, Florence Piron, Marc Rhainds, Martin Coulombe, Dolores Lepage-Savary, Marie Desmartis, Mylène Tantchou Dipankui, France Légaré

**Affiliations:** 1 Centre hospitalier universitaire de Québec (CHUQ) Research Centre, Hôpital Saint-François d'Assise, 10 rue de l'Espinay, D6-726, Quebec, QC, G1L 3L5, Canada; 2 Faculty of Nursing, Université Laval, Quebec, Canada; 3 Department of Management, Université Laval, Quebec, Canada; 4 National Collaborating Centre for Healthy Public Policy, Quebec, Canada; 5 Department of Information and Communication, Université Laval, Quebec, Canada; 6 Centre hospitalier universitaire de Québec, Quebec, Canada; 7 Institut National d'excellence en santé et en services sociaux, Quebec, Canada; 8 Department of Family Medicine, Université Laval, Quebec, Canada

**Keywords:** Health technology assessment, patient involvement, decision making, knowledge users, alternatives to isolation and restraint

## Abstract

**Background:**

The literature recognizes a need for greater patient involvement in health technology assessment (HTA), but few studies have been reported, especially at the local level. Following the decentralisation of HTA in Quebec, Canada, the last few years have seen the creation of HTA units in many Quebec university hospital centres. These units represent a unique opportunity for increased patient involvement in HTA at the local level. Our project will engage patients in an assessment being carried out by a local HTA team to assess alternatives to isolation and restraint for hospitalized or institutionalized adults. Our objectives are to: 1) validate a reference framework for exploring the relevance and applicability of various models of patient involvement in HTA, 2) implement strategies that involve patients (including close relatives and representatives) at different stages of the HTA process, 3) evaluate intervention processes, and 4) explore the impact of these interventions on a) the applicability and acceptability of recommendations arising from the assessment, b) patient satisfaction, and c) the sustainability of this approach in HTA.

**Methods:**

For Objective 1, we will conduct individual interviews with various stakeholders affected by the use of alternatives to isolation and restraint for hospitalized or institutionalized adults. For Objective 2, we will implement three specific strategies for patient involvement in HTA: a) direct participation in the HTA process, b) consultation of patients or their close relatives through data collection, and c) patient involvement in the dissemination of HTA results. For Objectives 3 and 4, we will evaluate the intervention processes and the impact of patient involvement strategies on the recommendations arising from the HTA and the understanding of the ethical and social implications of the HTA.

**Discussion:**

This project is likely to influence future HTA practices because it directly targets knowledge users' need for strategies that increase patient involvement in HTA. By documenting the processes and outcomes of these involvement strategies, the project will contribute to the knowledge base related to patient involvement in HTA.

## Background

The purpose of health technology assessment (HTA) is to summarize information about the clinical, economic, psychological, social and ethical aspects of health technologies (e.g. prevention programs, drugs, medical devices, procedures or systems used in screening, diagnosis, treatment, rehabilitation or palliation) in order to inform decisions about the introduction or use of these technologies [1]. To date, however, most HTA activities have focused on the clinical effectiveness and cost effectiveness of health technologies to the detriment of the other dimensions [2-4], such as their social and ethical aspects and the patient perspective, both of which are emphasized as important in the literature [5-11]. As direct beneficiaries of the technologies, patients have concrete knowledge of the impact and effects of treatments and technologies on their condition and on other areas of their lives [1]. Taking their perspective into account is therefore essential in order to arrive at a more complete assessment of the real value of health technologies and of their impact on the health of populations [7].

Policy makers, managers and HTA producers are thus increasingly interested in exploring strategies for incorporating the patient perspective in HTA activities [6,12]. A systematic review of the literature [13] assessing international studies in the area of patient and public involvement in HTA led us to observe that despite all the theoretical benefits commonly associated with involving patients and the public in HTA, there is little evidence to support these benefits. In fact, in the scientific literature [13], there are very few empirical evaluations of patient or public involvement in HTA. This lack of documentation may be due to the fact that patient involvement is seen more as an end in itself, rather than a means, and to the difficulty of evaluating their involvement without the support of widely accepted frameworks and tools [14].

Nonetheless, this systematic review identified 24 studies showing different means of integrating the patient perspective in assessing technologies or specific methods of dispensing and organizing services. Various experiences of patient consultation in HTA, such as the definition of schizophrenia treatment outcomes relevant to patients [15] or the analysis of the impact of pressure ulcers on quality of life [16], have been conducted by collecting primary research data. Some of these studies report that focusing on the patient perspective raises important issues, so far ignored, related to the use of assessed technologies. For instance, a study by Kinter [15] has shown that some of the criteria deemed essential by schizophrenic patients concerning their medical treatment (such as the ability to think clearly, participate in social activities, etc.) did not coincide with traditional clinical criteria used in the assessment of this treatment. Other studies listed in this review evaluated the direct participation of patients in HTA activities, notably those conducted by the National Institute for Health and Clinical Excellence (NICE), and the National Coordinating Centre for HTA (NCCHTA) in the United Kingdom [17-20]. These studies highlighted several elements of successful patient participation experiences, such as adequate preparation of participants, clear presentation of the topic of the meeting, and recruitment of people affected by the health technology assessed. Some barriers are also identified, such as problems related to recruitment, time, patient skills and their ability to participate, as well as the cultural differences among committee participants.

Moreover, our exploration of HTA stakeholders' views and practices during interviews conducted in a previous project revealed that there is agreement on the relevance of considering patient perspectives in HTA even though few experiments have yet been conducted at the local level (Gagnon et al., unpublished data). According to stakeholders, two methods were usually deemed relevant to seek the patient perspective in HTA: a) consulting patients or their close relatives as part of data collection, when the type of technology and issues assessed were appropriate for that, for example, when they have a great potential impact on patient's quality of life, and b) directly involving patient representatives in the assessment process, particularly during the three stages of: developing the assessment plan (or protocol), discussing the assessment report and recommendations, and disseminating results (Gagnon et al., unpublished data).

These interviews enabled us to identify the main factors facilitating or impeding each type of involvement according to the different stakeholders. We were thus able to outline the main elements of a reference framework for patient participation in HTA at the local level, an outline which forms the basis of this project.

This research project will complement a project prioritized by the Sectorial Table on HTA at the Réseau universitaire intégré de santé de l'Université Laval (RUIS-UL) in Quebec, Canada. After collecting information from member institutions in 2009, this sectorial table selected a question with supra-regional scope for HTA, namely, alternatives to isolation and restraint for hospitalized or institutionalized adults. The HTA unit at the Centre hospitalier universitaire de Québec (CHUQ) was mandated to carry out the assessment.

The aim of this research project is to use our framework to implement and then evaluate interventions involving patients in the assessment of alternatives to isolation and restraint for hospitalized or institutionalized adults.

### Research question and objectives

This research aims to answer the following questions: What are the impacts of targeted interventions to promote patient involvement in HTA activities, and how effective are they?

The objectives of this project are:

1) To validate the reference framework developed in our previous project and to explore the relevance and applicability of different models of patient involvement in the specific context of assessing alternatives to isolation and restraint for hospitalized adults or elderly people in nursing homes.

2) To implement interventions involving patients (including close relatives and representatives) at different stages of the mandated assessment process and also in the dissemination of its results.

3) To evaluate the intervention processes to further understand the contextual issues (ethical, social and organisational) of patient involvement in HTA activities.

4) To explore the impacts of these interventions on: a) the applicability and acceptability of recommendations arising from the assessment from the patients' perspective and that of the other groups involved in HTA; b) patient satisfaction; and c) the sustainability of this approach in HTA activities.

## Methods and design

This research project was designed with the collaboration of the HTA unit at the Quebec University Hospital Centre (Centre hospitalier universitaire de Québec - CHUQ) and its partners. We adopt an action research strategy which allows for constant adjustments between research objectives, methods, data collection, and analysis. The various activities we will undertake to achieve each objective of the study are described in the following section.

### Objective 1

#### Validate the reference framework and explore the relevance and applicability of patient involvement models

To achieve this objective, individual interviews will be conducted with representatives of the regional health agencies targeted by the project, with members of health and social services centres (CSSS) and HTA organisations, and with representatives of patient associations affected by the use of alternatives to isolation and restraint for hospitalized or institutionalized adults. We expect to carry out about 20 interviews in the entire area served by the RUIS-UL. An interview guide will cover questions on the applicability and relevance of the different models of patient involvement in the HTA of alternatives to isolation and restraint for hospitalized adults or elderly people in nursing homes. Interviews will be recorded digitally following informed consent of participants. The content of the interviews will be transcribed verbatim and analysed using N*Vivo software [21].

A thematic analysis of the content will be carried out using a three steps method described by Huberman and Miles [22]: data reduction, data display, and drawing conclusions/verification. To ensure the internal validity of the analysis process, interview codification will be carried out independently by two members of the team who have long experience in qualitative research using an iterative approach. The results will be then pooled and a consensus sought among the researchers with regards the final codification. Validating our reference framework this way is likely to result in the most appropriate strategies, methods and tools in the context of current practice in HTA, and will therefore have the most potential for sustainability.

### Objective 2

#### Development of interventions aimed at improving the participation of patients in HTA

Overall, four stages of the HTA process can be described [23] and subdivided into several sub-stages. The first is the selection of the technologies to be assessed and includes identifying and prioritizing the assessment topics. The second is conducting the assessment, including the following steps: development of the protocol (or assessment plan), review of the evidence-based data, contextualization of the data and a field assessment if relevant, analysis and synthesis of the results. The third stage is the production of a final report which may include recommendations, and the last stage of the HTA process is the dissemination and implementation of the results.

According to our systematic review, patient or public involvement is possible at each of these stages [13]. However, interviews conducted during the previous project enabled us to identify certain steps (corresponding to different sub-stages) that are particularly pertinent for patient involvement. These steps are: a) the development of the assessment plan (which allows patients' views and concerns to be considered in analysing the issues), b) the assessment itself (through consultation to collect data from patients about their experiences, perceptions and views), c) the discussion of the final report and recommendations, and d) the dissemination of the results [13]. Different modes of patient involvement (that correspond to different levels of involvement) may be used at these different steps [24] (see Additional File 1), mainly consultation - i.e data collection from patients - and direct participation of patients in the committees or working groups set up for a specific assessment.

Our project aims to implement and evaluate three strategies of patient involvement in the HTA process (See Additional File 1): 1) the direct participation of patient representatives in the HTA process of assessing alternatives to isolation and restraint for hospitalized or institutionalized adults; 2) the consultation, through data collection, of patients or/and their close relatives to inform the assessment; and 3) the involvement of patients in the dissemination of the HTA results.

For the first strategy, direct patient participation in the HTA process, patient representatives will be involved in a multidisciplinary working group specially established by the HTA unit for this assessment. The local HTA unit sets up a working group for every assessment it undertakes, but for this project the working group will be somewhat different from usual because of its supra-regional scope and because it involves representatives from several health regions. Patient representatives will be added to this working group to offer their perspective and their concrete experience as beneficiaries of the technologies assessed. They will be recruited through mental health and/or geriatric patients' associations. For the selection and supervision of these representatives, we will draw on the experience of other HTA agencies such as the National Institute for Health and Clinical Excellence (NICE) and the National Health Service (NHS) of the United Kingdom (INVOLVE, a national advisory group for promoting public involvement in NHS, public health and social care research) [25].

As for the second strategy, the consultation of patients (or their close relatives) through primary data collection will be conducted according to methods reported in the literature on similar subjects [2,15,16,26,27]. We plan to conduct about six (6) focus groups involving patients and/or their close relatives. These focus groups will encourage participants to discuss their experiences and perceptions of alternatives to isolation and restraint.

The third strategy, involving patients in the dissemination and communication of the HTA results, will be achieved in close collaboration with mental health and/or geriatric patients' associations involved in the HTA project. We will draw on the experience of the NHS Centre for Reviews and Dissemination in the United Kingdom [28] for the production of written material suitable for members of these associations. The focus groups will be consulted to make sure that the results presented are accessible and useful to patients. The project team researchers will collaborate with representatives of target groups who have experience preparing materials appropriate for communicating the results of the assessment.

Patient representatives will also be asked to play the role of ambassadors in "translating" the recommendations in the final HTA report so they can be transmitted back to their associations and to the various mental health and geriatric forums.

To improve our chances for successful interventions, our approach will be based on the best scientific evidence available about effective strategies for patient involvement [13] and on the reference framework we developed in collaboration with HTA producers and HTA users [29].

### Objectives 3 and 4

#### Evaluation of the process and impact of patient involvement

The strategies used in this project must be evaluated to establish their practicability and to provide evidence-based data that will inform future strategies for involving patients in HTA [30]. As this study is likely to influence practices in HTA, evaluation of the effectiveness of our strategies will focus particularly on how the patient involvement process is set up and subsequently followed up, taking the views of all the different participants into account. We will also evaluate the impact of our patient involvement strategies on the recommendations arising from the HTA, and evaluate patient understanding of the ethical and social issues relating to the HTA.

We will base our evaluation of strategies on two sets of criteria, those related to the process and those associated with results and impacts. We will develop our evaluation plan from the model proposed by Rowe [31] that identifies nine criteria for assessing patient involvement: five related to the effective construction and implementation of a procedure (representativeness, independence, early involvement, influence or impact on decision making, and transparency) and four related to process (accessibility of resources, task definition, exercise of structured decision making, and cost-effectiveness). However, a recent analysis by Rowe and collaborators [14] of these criteria showed that although they have a certain validity, they are not exhaustive nor necessarily appropriate for all involvement activities or all contexts. We will consequently use additional criteria, some based on information we gathered during interviews with various actors in HTA in Quebec [32] and others that we identified from the literature on patient and public participation.

The choice of outcome indicators, methods and analysis strategies will be based on discussions with researchers and policy makers, an approach recognized as being the most effective for knowledge translation [33,34]. The research team will observe and evaluate the process and results of the patient involvement strategies at each stage of the project. The evaluation will be participatory [35-38], which means that it will involve stakeholders at each step of the evaluation (planning, design, data collection and analysis, identification of findings, conclusions, recommendations and dissemination of results).

At the beginning of the project a workshop will be conducted with knowledge users and team researchers to reach a consensus on the objectives and evaluation questions. The data collection methods, the outcome indicators, the data analysis plan, and the roles and responsibilities of each team member will also be discussed. This collaborative approach is likely to increase our success in strategy implementation, as the results will relate directly to the challenges knowledge users face and will address their practical needs. They will thus be more likely to follow the recommendations when making decisions [39].

The evaluation of involvement strategies will be based on a mixed-method approach. Semi-structured interviews with various stakeholders (patient representatives, HTA producers, and healthcare managers) will be held after each phase of the project to identify the benefits and drawbacks of each strategy, the factors that eased or complicated its implementation, as well as how the strategy influenced the HTA process, the recommendations, and the perceptions of the various stakeholders. The evaluation will also address the questions of time required, human and material resources needed, and associated costs. This will provide data for knowledge users interested in implementing similar strategies. The evaluation will also focus on the possibility of using the tested strategies in other HTA projects. The proposed approach is thus in keeping with the knowledge translation cycle [40] in that the knowledge produced and its evaluation within one context will serve as a basis for developing interventions in other contexts aiming at greater involvement of patients in HTA.

### Knowledge translation activities

The two elements most often associated with the usefulness of knowledge emerging from research findings in decision making are the timeliness of the data and the ongoing relationships between researchers and policy makers [41]. Our approach to knowledge translation (KT) is therefore based on these two keys elements. First, the project answers a need expressed by policy makers and HTA producers to move towards greater involvement of patients in HTA activities. Second, the close collaboration between researchers and knowledge users in the team, which began with a previous project and has been nourished by many exchanges since, will continue throughout this project. Thus the participatory approach [41] characterizing all stages of this research project will promote mutual ownership of the research results.

To facilitate the translation of our results we will organize presentations in institutions involved in HTA in the RUIS-UL. Presentations will also be offered to a wider audience (such as the Ministry of Health and Social Services) so that they can validate our process and results and we can assess their applicability to other organizations interested in HTA. These presentations will form an integral part of the regular training activities of these organizations. To ensure their effectiveness, we will target key messages identified during workshops we previously conducted with policy makers and patients to the specific audiences (managers, health professionals, HTA producers and patients). The presentations could also be made by knowledge users belonging to the team or by patient representatives or researchers, depending on the target audience. Thus, an interactive process of knowledge translation will promote participation of targeted knowledge users (44, 45). Towards the end of the project, we will organize a provincial meeting for representatives of the main HTA producers and users groups (policy makers, managers, HTA producers, health professionals and associations of patients).

## Discussion

A previous systematic review found little evidence about effective strategies to involve patient in HTA [13]. In the field of clinical practice guidelines, a systematic review found that patient and public involvement was more frequent in mental health [42]. This project represents a unique opportunity for policy makers and researchers to work toward a common goal to test different forms of patient involvement in HTA and to evaluate them in order to produce useful knowledge regarding the implementation of patient involvement strategies in HTA and their possible effects and impacts. By creating links between research and practice, this project will establish a good balance between the interests and the expertise of researchers and those of knowledge users. The knowledge users involved are already working with researchers on the team; our collaborative approach will ensure a common understanding of the project's objectives, activities and results [33,34]. This will facilitate the translation of knowledge that is tailored to the local context and to the specific reality of knowledge users.

Since this study meets the needs expressed by knowledge users belonging to the team and their partners in the RUIS-UL, the knowledge produced will be directly useful to guide practice in terms of patient involvement in HTA activities. In addition to answering a real need among decision makers and HTA knowledge users, this project will not only contribute to the advancement of knowledge about patient involvement strategies for HTA, but it will also foster a greater involvement of patients themselves in the field.

## Ethical considerations

Interviewees and participants in the focus groups will be asked to complete a consent form presenting research objectives and information about research implications. Ethics approval for the project has been received from the Research Ethics Board of the Centre Hospitalier Universitaire de Québec (approved on June 22, 2011; ethics number S11- 06-039).

## Competing interests

The authors declare that they have no competing interests.

## Authors' contributions

All authors collectively drafted the research protocol and approved the final manuscript. MPG is its guarantor.

## Pre-publication history

The pre-publication history for this paper can be accessed here:

http://www.biomedcentral.com/1472-6963/12/14/prepub

## Supplementary Material

Additional file 1 **Appendix 1**. Activities involving patients in phases of the assessment process in local HTA and levels of patient involvement. Click here for file
