# Supplementary material for: Involving patients in HTA activities at local level: a study protocol based on the collaboration between researchers and knowledge users
Source: BMC Health Serv Res. 2012 Jan 16;12:14. doi: 10.1186/1472-6963-12-14 (PMC3274445; doi:10.1186/1472-6963-12-14)
Supplement: Additional file 1 — Appendix 1. Activities involving patients in phases of the assessment process in local HTA and levels of patient involvement. [file 1472-6963-12-14-S1.DOC]

Appendix 1. Activities involving patients in phases of the assessment process in local HTA and levels of patient involvement

|  | **Phases of the assessment process** | **Activities involving patients** | **Levels of the involvement[[1]](#footnote-2)/ type of participants** |
| --- | --- | --- | --- |
| **SELECTION OF ASSESSMENT TOPICS** | **Submission of assessment requests** | No involvement of patients: subject prioritized by the Sectorial Table on HTA at the Réseau universitaire intégré de santé de l’Université Laval (RUIS-UL) after collecting information from member institutions. | |
|  | **Prioritization of requests** |  |  |
| **ASSESSMENT** | **Development of assessment plan** | Direct participation in the working group set up for the assessment | **Participation**/ Patient representatives |
|  | **Collection of evidence (Literature)** | No involvement of patients: literature review carry out by the HTA unit at the Centre hospitalier universitaire de Québec (CHUQ) and its partners | |
|  | **Contextualisation or collection of field data** | Focus groups to collect information or opinions about the topic | **Consultation /**Patients or service users or their close relatives |
|  | **Analyses and synthesis of results (first version of the report)** | First results will be presented at the working group that included patients members | **Participation** / Patient representatives |
| **REPORT AND RECOMMENDATIONS** | **Discussion and approval of the final report** | Direct participation in the working group set up for the assessment | **Participation /** Patient representatives |
|  | **Making recommendations** |  |  |
| **DIFFUSION** | **Diffusion of the report and recommendations** | Focus groups to design materials to communicate the results of the assessment to patients  Communication of the results of the assessments | **Participation /**Patient representatives  **Information/** patients and their representatives |

1. **Levels of involvement :** **Information**: to inform and raise awareness among patients

   **Consultation** : to collect information or opinions from patients **Participation** : to involve patients actively as partners [↑](#footnote-ref-2)
